# Surface display of an anti-DEC-205 single chain Fv fragment in *Lactobacillus plantarum* increases internalization and plasmid transfer to dendritic cells in vitro and in vivo

**DOI:** 10.1186/s12934-015-0290-9

**Published:** 2015-07-04

**Authors:** Michon Christophe, Katarzyna Kuczkowska, Philippe Langella, Vincent G H Eijsink, Geir Mathiesen, Jean-Marc Chatel

**Affiliations:** INRA, UMR1319 MICALIS, Bat 440, R-2, 78352 Jouy-en-Josas, France; AgroParisTech, UMR MICALIS, 78352 Jouy-en-Josas, France; Department of Chemistry, Biotechnology and Food Science, Norwegian University of Life Sciences, Aas, Norway

**Keywords:** Dendritic cell, DEC-205, Lactic acid bacteria, Drug delivery, Cellular targeting, Surface display

## Abstract

**Background:**

Lactic acid bacteria (LAB) are promising vehicles for delivery of a variety of medicinal compounds, including antigens and cytokines. It has also been established that LAB are able to deliver cDNA to host cells. To increase the efficiency of LAB-driven DNA delivery we have constructed *Lactobacillus plantarum* strains targeting DEC-205, which is a receptor located at the surface of dendritic cells (DCs). The purpose was to increase uptake of bacterial cells, which could lead to improved cDNA delivery to immune cells.

**Results:**

Anti-DEC-205 antibody (aDec) was displayed at the surface of *L. plantarum* using three different anchoring strategies: (1) covalent anchoring of aDec to the cell membrane (Lipobox domain, Lip); (2) covalent anchoring to the cell wall (LPXTG domain, CWA); (3) non-covalent anchoring to the cell wall (LysM domain, LysM). aDec was successfully expressed in all three strains, but surface location of the antibody could only be demonstrated for the two strains with cell wall anchors (CWA and LysM). Co-incubation of the engineered strains and DCs showed increased uptake when anchoring aDec using the CWA or LysM anchors. In a competition assay, free anti-DEC abolished the increased uptake, showing that the internalization is due to specific interactions between the DEC-205 receptor and aDec. To test plasmid transfer, a plasmid for expression of GFP under control of an eukaryotic promoter was transformed into the aDec expressing strains and GFP expression in DCs was indeed increased when using the strains producing cell-wall anchored aDec. Plasmid transfer to DCs in the gastro intestinal tract was also detected using a mouse model. Surprisingly, in mice the highest expression of GFP was observed for the strain in which aDec was coupled to the cell membrane.

**Conclusion:**

The results show that surface expression of aDec leads to increased internalization of *L. plantarum* and plasmid transfer in DCs and that efficiency depends on the type of anchor used. Interestingly, in vitro data indicates that cell wall anchoring is more effective, whereas in vivo data seem to indicate that anchoring to the cell membrane is preferable. It is likely that the more embedded localization of aDec in the latter case is favorable when cells are exposed to the harsh conditions of the gastro-intestinal tract.

## Background

DNA vaccination relies on the injection of a plasmid containing cDNA under control of an eukaryotic promoter into host cells. It has been shown that DNA vaccination is able to elicit immune responses comparable to the responses generated by attenuated pathogens [1]. DNA vaccines can be administered by intramuscular injection of DNA, but this strategy needs trained staff and equipment, and may in some cases suffer from DNA instability [2]. Mucosal delivery of the vaccines is of interest because it can elicit both local and systemic immunization. Because DNA is very sensitive to the physico-chemical conditions in the gut, the use of DNA vaccination at the mucosal level implies the development of vectors able to protect the DNA. Alternatives include encapsulation of the cDNA in nanoparticles of polysaccharides or in liposomes, as well as the use of bacterial vectors [3–5].

Lactic acid bacteria (LAB) including *Lactobacillus*, *Lactococcus*, *Streptococcus* or *Enterococcus* are Gram positive bacteria. LAB are present in a wide range of ecological niches such as plant material and the gastro intestinal tract (GIT) and they have been used for thousands years in fermented food products. LAB have been given the GRAS (generally recognized as safe) status by the World Health Organization and several LAB strains have probiotic properties. Probiotic effects of LAB have been shown to be beneficial in relation to lactose intolerance [6], diarrhea [7], allergy [8, 9], inflammatory bowel disease (IBD) [10] and cancer [11]. Notably, the beneficial effects of LAB include immunomodulatory effects and an adjuvant-type of action [12]. One of the LAB for which such effects have been studied in most detail is *Lactobacillus plantarum* [12] [8] [13]. Importantly, LAB resist low pH and the harsh conditions in the GIT, making them a vector of choice for oral administration.

The possibility of LAB delivering cDNA to host cells has proven its efficiency in several applications. The first demonstration concerned a *Lactococcus lactis* strain carrying cDNA encoding β-lactoglobulin (BLG), one of the major allergens in milk [14, 15]. Recently, it was shown that *L. lactis* carrying cDNA encoding IL-10 under control of the eukaryotic cytomegalovirus (CMV) promoter has protective effects in a mouse model of trinitrobenzene sulfonic (TNBS) acid-induced colitis [16]. Delivery of a DNA vaccine by *L. acidophilus* had a positive effect in protecting mice from foot and mouth disease [17].

Enhancing interactions between host cells and LAB may be one way to increase the transfer efficiency of cDNA-based vaccines, and therefore, several studies aimed at targeting specific cellular populations have been performed. For example, fibronectin binding protein A (FnBPA) from *Staphylococcus aureus* and mutated internalin A (InlA) from *Listeria monocytogenes* have been successfully expressed at the surface of *L. lactis* [18, 19]. These two proteins are responsible for binding of *S. aureu*s and *L. monocytogenes*, respectively, to epithelial cells by interacting with specific receptors [20, 21]. Binding is the first step towards internalization and it was indeed shown that transfer of a plasmid expressing either GFP or BLG was enhanced when LAB expressed FnBPA or mutated InlA [18, 19].

Dendritic cells (DCs) are antigen presenting cells and thus major players in the immune response. They are present in tissue mucosa, under the epithelial barrier of the gut, and they can extend dendrites through tight junctions to sample antigens in the lumen [22]. DCs are an important link between the exterior and the immune system. They have numerous different pattern recognition receptors (PRRs) and when they encounter a potential pathogen, they switch to an activated state [23]. Once activated, they migrate to lymph nodes where they stimulate T cells proliferation and differentiation.

One of the PRRs present in DCs is DEC-205 (or CD-205) which is a C-type lectin receptor involved in recognition of ligands expressed during apoptosis and necrosis of cells [24], in recognition of CpG oligonucleotides [25] and in antigen processing [26]. This receptor has been a target in several vaccine improvement studies [27]. For example, tumor associated antigen HER2/neu has been fused to a single chain Fv fragment (ScFv) targeting DEC-205. Administration of the fusion protein elicited a higher cellular and humoral response and had a stronger protective effect against tumor formation, compared to the antigen alone [28]. DEC-205 is also a target to induce tolerance to substances. For example, Bruder et al. [29] showed that fusing a tolerogenic vaccine against type 1 diabetes to an anti-DEC-205 antibody protected mice from developing this autoimmune disease. These effects are likely due to the fact that when an antigen is endocytosed via DEC-205, it is presented to the immune system through both MHC class I and MHC class II molecules, thus to both CD4+ and CD8+ T cells [30].

In the present study, we investigated the potential of targeting DEC-205 as a tool to enhance interactions between LAB and DCs and thus plasmid DNA delivery. We have constructed strains of *L. plantarum* expressing a recombinant ScFv against murine DEC-205 (aDec) at their surface, using three different surface anchors. We show that surface localization of aDec in *L. plantarum* has the potential to increase internalization of the bacterium and plasmid transfer both in cell culture and in mice and that this potential depends on the type of surface anchor.

## Results

### Construction of recombinant *Lactobacillus plantarum* strains producing the anti-DEC-205 ScFv

**Figure 1:**
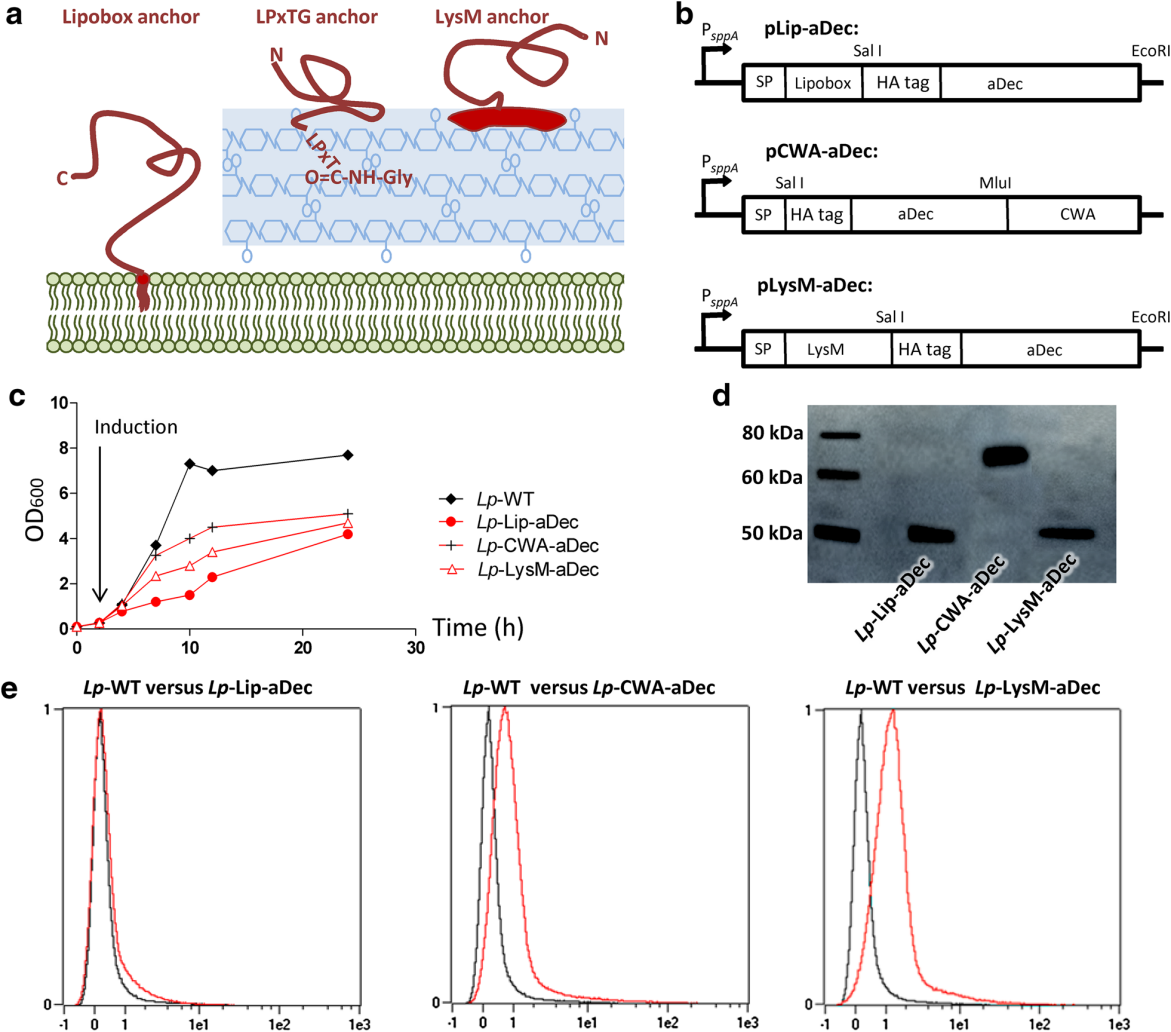
Characterization of aDec-expressing *L. plantarum* strains. **a** Schematic representation of the three anchors used. **b** Expression cassettes in which the gene fragment encoding the desired protein is translationally fused to the inducible P_*sppA*_ promoter [47]. The cassette was PCR-generated using the primers listed in Table 2 and inserted into previously described anchoring vectors [31, 36] digested with the restriction enzymes indicated in the *Figure*, as summarized in Table 3. All constructs include a N-terminal signal sequence (SP) for secretion and a HA-tag for immune detection. Three different anchoring domains were used, as described in the *main text*. Note that in the CWA construct, the anchoring domain is located C-terminally, meaning that the HA-tag will protrude from the cells after secretion and subsequent anchoring. **c** Growth curves for *Lp*-WT (control strain) and aDec-expressing strains; protein production was induced by addition of peptide pheromone at OD600 ~0.3. **d** Western blot analysis of cell-free protein extracts from aDec-expressing strains harvested 2 h after induction, using a mouse anti-HA primary antibody and a HRP-conjugated goat anti-mouse secondary antibody. **e** Flow cytometry analysis of the presence of aDec at the surface of *Lp*-WT (*in black*) compared to *Lp*-Lip-aDec, *Lp*-CWA-aDec or *Lp*-LysM-aDec (*all in red*).

A DNA fragment encoding the anti-mouse Dec205 ScFv (aDec) preceded by a HA-tag for immune detection was cloned into plasmids previously developed for production and surface localization of proteins in *L. plantarum* (32, 26) using three different types of anchors as outlined in Figure 1a, b. The anchors are: a Lipobox membrane anchor for covalent coupling of aDec to a membrane component, a cell wall anchor based on sortase-catalyzed covalent coupling to the peptidoglycan and a cell wall anchor based on non-covalent interactions between a LysM domain and the cell wall. The three resulting plasmids, pLip-aDec, pCWA-aDec and pLysM-aDec (Figure 1b) were transformed into *L. plantarum*. In addition, we used *Lp*-WT, containing a control plasmid (pEV) [31] that does not encode for the anchors or aDec, as a negative control. The growth rate of strains producing aDec was substantially lower compared to control strain (Figure 1c), in particular for the strain harboring the lipoprotein anchor. Still, all strains showed reasonable growth and bacteria harvested 2 h after induction, where all transformants have a similar OD_600_ (Figure 1c) and similar cell counts, were used for further studies.

Western blot analysis of protein extracts of induced strains using anti-HA antibodies revealed the presence of HA tagged proteins with correct molecular size in all three aDec-producing strains (Figure 1d). To check surface localization we labeled the strains with a fluorescent anti-HA antibody and tested antibody binding by flow cytometry. *Lp*-CWA-aDec and *Lp*-LysM-aDec, but not *Lp*-Lip-aDec showed higher fluorescence than *Lp*-WT (Figure 1e). Taken together these results show that aDec is expressed in all three aDec-producing strains. However, aDec is only detectable at the surface in the two strains where aDec is fused to the cell wall anchors.

### Internalization of aDec-displaying *L. plantarum* by dendritic cells

**Figure 2:**
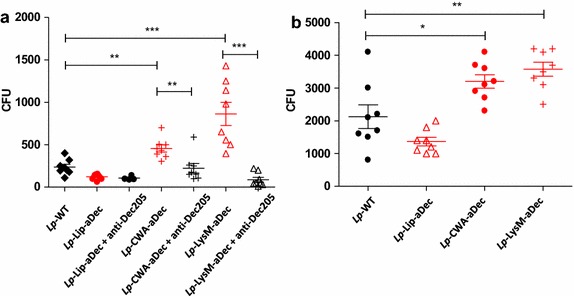
Effect of surface expression of aDec on internalization of *L. plantarum* by DCs. **a** Monocyte derived hDCs were co-incubated with *Lp*-WT, *Lp*-Lip-aDec, *Lp*-CWA-aDec or *Lp*-LysM-aDec with or without addition of a free anti human DEC205 antibody. After incubation, the hDCs were washed and non-internalized bacteria were killed by treatment with gentamicin. The numbers of internalized bacteria were counted as CFU. Same amount of DCs was used in each experiment. **b** BMDCs were co-incubated with *Lp*-WT, *Lp*-Lip-aDec, *Lp*-CWA-aDec or *Lp*-LysM-aDec. After incubation, the BMDCs were washed and non-internalized bacteria were killed by treatment with gentamicin. The numbers of internalized bacteria were counted as CFU. *Each point* represents independent wells and results are presented as mean ± SEM. The results presented are from one experiment representative of three performed independently. Statistically significant differences are indicated as follows: *p < 0.05; **p < 0.01; ***p < 0.001.

To test interactions of the recombinant strains with DCs derived from humans (hDCs) or mice bone marrow dendritic cells (BMDCs), we first performed internalization assays. Figure 2 shows that the number of internalized bacteria was significantly higher after co-incubation with *Lp*-CWA-aDec and *Lp*-LysM-aDec compared to *Lp*-WT or *Lp*-Lip-aDec. To investigate the specificity of the interactions between the displayed anti-mouse ScFv and human DCs, a competition experiment was carried in which the hDCs were pre-treated with a monoclonal antibody against human DEC-205. The results (Figure 2a) clearly show that increased internalization caused by surface display of aDec is abolished in the presence of the competing free antibody, which proofs that increased internalization is due to a specific interaction between the recombinant strains and DEC-205 on the DCs.

### Effect of aDec expression on plasmid transfer to dendritic cells

**Figure 3:**
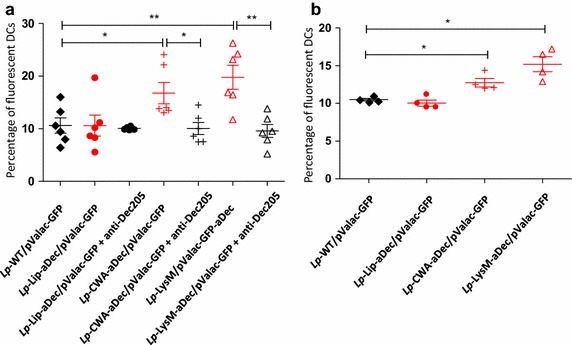
Effect of surface expression of aDec on plasmid delivery into DCs. **a** Monocyte derived hDCs were co-incubated with *Lp*-WT/pValac-GFP, *Lp*-Lip-aDec/pValac-GFP, *Lp*-CWA-aDec/pValac-GFP or *Lp*-LysM-aDec/pValac-GFP with or without addition of a free anti-human DEC205 antibody. After incubation the percentage of green fluorescent DCs was determined by flow cytometry. **b** BMDCs were co-incubated with *Lp*-WT/pValac-GFP, *Lp*-Lip-aDec/pValac-GFP, *Lp*-CWA-aDec/pValac-GFP or *Lp*-LysM-aDec/pValac-GFP. After incubation, the percentage of fluorescent DCs was determined by flow cytometry. *Each point* represents independent wells and the results are presented as mean ± SEM. The results presented are from one experiment representative of three performed independently. Statistically significant differences are indicated as follows: *p < 0.05; **p < 0.01; ***p < 0.001.

To investigate the potential of the recombinant strains for plasmid transfer we transformed the strains with an additional plasmid, pValac-GFP [32] for expression of GFP cDNA under the control of the eukaryotic CMV promoter. We thus obtained *Lp*-WT/pValac-GFP, *Lp*-Lip-aDec/pValac-GFP, *Lp*-CWA-aDec/pValac-GFP and *Lp*-LysM-aDec/pValac-GFP (Table 1). Growth of the pValac-GFP containing double transformants was similar to growth of the corresponding single transformants depicted in Figure 1c. Figure 3a shows that the number of hDCs expressing GFP was significantly higher after co-incubation with *Lp*-CWA-aDec/pValac-GFP or *Lp*-LysM-aDec/pValac-GFP compared to *Lp*-WT/pValac-GFP or *Lp*-Lip-aDec/pValac-GFP. Similar results, although with lower significance, were obtained with BMDCs (Figure 3b). Competition experiments with the free anti DEC-205 antibody showed that increased plasmid transfer is due to a specific interaction between the recombinant strains and DEC-205 on the DCs.

**Table 1:** Bacterial strains and plasmids used in this study

Designation	Strains	Characteristic	References
	*Lb. Plantarum* WCFS1	Host strain	[34]
	*E. coli* TOP10	Host strain	Invitrogen
	*Lp*-WT	WCFS1 with pEV	This study
	*Lp*-WT/pValac-GFP	WCFS1 with pEV and pValac-GFP	This study
aDec strains	*Lp*-Lip-aDec	WCFS1 with pLip-aDec	This study
	*Lp*-CWA-aDec	WCFS1 with pCWA-aDec	This study
	*Lp*-LysM-aDec	WCFS1 with pLysM-aDec	This study
	*Lp*-Lip-aDec/pValac-GFP	WCFS1 with pLip-aDec and pValac-GFP	This study
	*Lp*-CWA-aDec/pValac-GFP	WCFS1 with pCWA-aDec and pValac-GFP	This study
	*Lp*-LysM-aDec/pValac-GFP	WCFS1 with pLysM-aDec and pValac-GFP	This study

Designation	Plasmids	Characteristic	References
	pEV	Control plasmid	[31]
aDec plasmids	pLip-aDec	Plasmid expressing aDec with lipoanchor	This study
	pCWA-aDec	Plasmid expressing aDec with LPXTG anchor	This study
	pLysM-aDec	Plasmid expressing aDec with LysM anchor	This study
	pValac-GFP	Plasmid expressing GFP under CMV promoter	[32]

### Plasmid delivery from bacteria to dendritic cells in vivo

**Figure 4:**
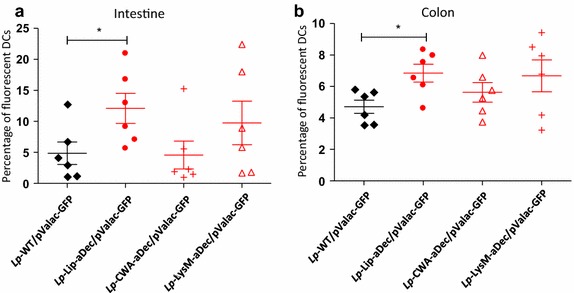
Effect of surface expression of aDec on plasmid transfer to mouse DCs in vivo. Mice were orally administrated with *Lp*-WT/pValac-GFP, *Lp*-Lip-aDec/pValac-GFP, *Lp*-CWA-aDec/pValac-GFP or *Lp*-LysM-aDec/pValac-GFP during 4 days. At day 4 mice were sacrificed and DCs were extracted from the small intestine (**a**) or the colon (**b**). The percentage of DCs expressing GFP was measured by flow cytometry. *Each point* represents independent mice and the results are presented as mean ± SEM. Statistically significant differences are indicated as follows: *p < 0.05; **p < 0.01; ***p < 0.001.

To investigate the potential of the recombinant strains for plasmid transfer in vivo the recombinant strains containing pValacGFP were administered to mice. After 4 days of oral administration of the four different strains, mice were euthanized and DCs were isolated from the intestine and the colon. Quantification of the fraction of fluorescent DCs by flow cytometry showed that, compared to mice fed with control strain, this fraction was higher in both the intestine (Figure 4a) and the colon (Figure 4b) for mice fed with *Lp*-Lip-Dec/pValac-GFP. A similar trend, but not significant, was observed for mice fed with *Lp*-LysM-aDec/pValac-GFP. Thus, expression of aDec at the membrane of *L. plantarum* enhances plasmid transfer in vivo, but the preferred anchor (Lip) differs from the anchors that were most efficient in vitro (CWA and LysM).

## Discussion

In the last decade promising results have been reported concerning the potential of LAB for drug delivery [33]. One of the most thoroughly studied LAB is *L. plantarum* whose genome was one of the first bacterial genomes to be sequenced [34]. Some *L. plantarum* strains have been described as probiotics [12] and the persistence of these species in the GIT is a main advantage for drug delivery. Several previous studies have shown that *L. plantarum* can be modified to express a variety of medicinally important proteins and that surface anchoring can be achieved in various manners [35, 36]. Importantly, a previous study on anchoring invasin on the surface of *L. plantarum* showed that the functionality of the invasin-expressing strains depended on the type of anchor used [31]. Here, we have used these previously described anchors to explore a strategy for targeting DCs, with the ultimate goal of using this novel trait to develop more efficient LAB-based DNA vaccines.

Three surface-anchoring strategies were used to display aDec, an ScFv fragment binding to DEC-205, a C-type lectin receptor of the DCs membrane (Figure 1). Western blotting showed that the antibody fragment was indeed produced in all three corresponding strains, but in subsequent flow cytometry analyses it was only detected at the surface in two of these. The lack of detection of aDec at surface of the *Lp*-Lip-aDec strain, despite the protein being highly expressed (Figure 1d), is most likely caused by the protein being embedded in the cell wall. The localization of the HA-tag at the very N-terminus of the protein, i.e. directly after the lipo-anchor, may preclude its detection with the anti-HA antibody.

In order to test the effects of our surface display of an anti-DEC205 ScFv on bacteria-DCs interactions, we used two sources of DCs, murine BMDCs and human DCs derived form monocytes which are known to express DEC205 at their surface [37] [38]. Internalization assays showed that surface display of aDec indeed increases internalization by human DCs and, to a lesser extent, murine BMDCs. Internalization by DCs was significantly higher for *Lp*-CWA-aDec and *Lp*-LysM-aDec strains compared to *Lp*-WT and the lipoprotein construct *Lp*-Lip-aDec. The lack of internalization of *Lp*-Lip-aDec could be due to limited accessibility of aDec, as alluded to above. Importantly, competition assays with free antibodies confirmed that increased internalization indeed is due to specific interactions of the recombinant lactobacilli with DEC-205. This shows that *L. plantarum* is able to produce at its surface a fully functional recombinant antibody, which leads to increased internalization of the recombinant bacteria by the DCs.

One of the aims of the present study was to enhance the internalization efficiency of bacteria in DCs and, in addition, to optimize transfer of a functional plasmid into the DCs. To test this, we transformed the three aDec producing strains and the control strain with pValac-GFP, a plasmid previously described containing an expression cassette with GFP cDNA under the control of an eukaryotic promoter [15]. DCs co-incubated with *Lp*-CWA-aDec/pValac-GFP or *Lp*-LysM-aDec/pValac-GFP showed significantly higher fractions of GFP expressing cells compared to DCs co-incubated with *Lp*-Lip-aDec/pValac-GFP or *Lp*-WT-aDec/pValac-GFP. Thus, those strains in which the highest levels of surface-located aDec were detected also showed the highest levels of internalization. We have previously observed a correlation between the extent of internalization of bacteria and the extent of plasmid transfer [39]. For example expression of FnBPa or InlA at the surface of *L. lactis* increased internalization in Caco-2 cells and enhanced plasmid transfer both in vitro and in vivo [40–42].

To test the uptake and transfer of the GFP encoding plasmid in vivo, mice were orally administrated with relevant strains and plasmid transfer was monitored by GFP expression in DCs extracted from the GIT. Surprisingly, we observed significantly higher expression of GFP in DCs from mice administrated with *Lp*-Lip-aDec/pValac-GFP compared to other strains. The same trend was observed in mice fed with *Lp*-LysM-aDec/pValac-GFP, but in this case the increase was not significant. Thus, the strain that showed good protein expression but that seemed least efficient in terms of surface display and in vitro internalization and plasmid transfer gave the best in vivo results. Another factor potentially playing a role could be that the (harsh) conditions in the gut change the cell wall of the bacteria, which somehow could promote exposure of cell-wall embedded Lip-aDec.

Several studies, mainly using nanoparticles, have shown the efficiency of targeting a vaccine to DCs using aDec. For example, liposomes or bacteriophages displaying anti-DEC-205 and producing the OVA antigen induced protective effects against highly metastatic murine melanoma (B16-OVA) in mice [43, 44]. Importantly, use of DEC-205 targeting nanoparticles was shown to be more efficient than administrating nanoparticles without targeting molecules [45]. The present study adds LAB as a possible DEC-205 targeting delivery vehicle. Existing data show the possibility of replace the model GFP gene used here by other medically important genes.

## Conclusions

We show that *L. plantarum* is able to produce and surface display a fully functional recombinant antibody, aDec, and thus target the DEC-205 receptor, which results in increased internalization of the bacteria. The results further show that the functionality of the aDec-expressing strains depends on the type of anchor used. Expression of surface-located aDec increases plasmid transfer from bacteria to DCs in vitro and in the GIT. The data indicate that to achieve efficient internalization in the GIT, a more embedded and protected localization of aDec is required.

## Methods

### Bacterial strains and induction

The bacterial strains and plasmids used in this study are listed in Table 1. *Escherichia coli* TOP10 cells (Invitrogen) were grown in brain heart infusion (BHI) broth (Oxoid) at 37°C with shaking*. L. plantarum* cells were grown statically in MRS broth (Oxoid) at 37°C. Solid media were prepared by adding 1.5% (w/v) agar to the broth. Plasmid constructs were first established in *E. coli* cells and subsequently after transformed into electrocompetent *L. plantarum* cells as described previously [46]. The antibiotic concentrations used were 5 and 200 µg/mL of erythromycin for *L. plantarum* and *E. coli*, respectively and 10 µg/mL of chloramphenicol. Induction of protein expression was done by adding the inducing peptide pheromone (SppIP) to a final concentration of 25 ng/mL at OD_600_ = 0.3, according to Sørvig et al. [47].

### DNA manipulations and plasmid construction

DNA manipulations were performed essentially as previously described [48]. The primers used in this study were purchased from Operon Biotechnologies and are listed in Table 2. We used the sequence of a single chain anti mouse DEC-205 Fv fragment [49] to design a synthetic gene, codon optimized for *L. plantarum* expression, comprising the Fv fragment with an N-terminal HA (hemagglutinin) tag (YPYDVPDYA), which was produced by Geneart (Lifetech). The plasmid containing the synthetic gene was then used as template in subsequent PCR reactions using hot start KOD polymerase (Toyobo). Amplified PCR fragments were separated on 1% agarose gels and purified using the NucleoSpin extract II kit (Macherey–Nagel). After purification the PCR fragments were cloned into restriction-digested plasmids containing various secretion and anchoring signals [31, 36] using the In-Fusion HD cloning kit (Clontech Laboratories), following the manufacturer’s instructions. Vectors, primers and restriction enzymes used in cloning are summarized in Table 3 and essential aspects of the constructs are displayed in Figure 1b. Plasmid DNA was purified from *E. coli* using the NucleoSpin plasmid kit (Macherey–Nagel GmbH & Co). Competent *L. plantarum* were transformed by electroporation according to a previously described method [46]. The DNA sequences of all PCR amplicons were verified by sequence analysis.

**Table 2:** List of PCR primers used in this study

Primer name	Sequence
LipaDecFW	GATTGCGGCGGTCGACTATCCATATGATGTTCCAGATTATGC
LipaDecBW	CCGGGGTACCGAATTCTTACGATATCCCTGATGAAACTGTAAC
CwaaDecFW	TGCTTCATCAGTCGACTATCCATATGATGTTCCAGATTATGC
CwaaDecBW	GTTCAGTGACACGCGTCGATATCCCTGATGAAACTGT
LysMdecFW	TTGGGCCCTTGTCGACTATCCATATGATGTTCCAGATTATGC
LysMdecBW	CCGGGGTACCGAATTCTTACGATATCCCTGATGAAACTGTAAC

**Table 3:** Method for plasmid construction

Plasmid name	Original plasmid	References	Restriction sites	Primer used for aDEC amplification
pLip-aDec	pLp_1452Inv	[31]	*Sal*I, *Eco*RI	LipaDecFW, LipaDecBW
pCWA-aDec	pLp_0373sOFAcwa2	[36]	*Sal*I, *Mlu*I	CwaaDecFW, CwaaDecBW
pLysM-aDec	pLp_3014Inv	[31]	*Sal*I, *Eco*RI	LysMdecFW, LysMdecBW

### Protein extraction and western blot

The bacteria were harvested 2 h after induction and washed twice with ice-cold Tris-buffered sucrose (pH 7.0, 10 mM MgCl_2_, 250 mM sucrose). After centrifugation, the bacteria were disrupted by glass beads (106 micron, Sigma) using a FastPrep^®^-24 instrument (MP Biomedicals) to obtain protein extracts. After cell disruption and centrifugation, cell-free supernatants were mixed with SDS-PAGE sample buffer. After boiling the samples for 7 min, they were applied to 10% Stain-Free gels (Biorad), followed by electrophoresis with subsequent immunoblotting using the iBlot system (Thermo Fisher Scientific) according to the instructions of the manufacturer. Protein detection was performed with the SNAP i.d. System (Millipore), using a murine anti-HA antibody (Sigma) and a horseradish peroxidase (HRP)-conjugated goat anti-mouse antibody (BioRad). Proteins were visualized using the SuperSignal West Pico chemiluminescent substrate (Pierce).

### Detection of aDec at the surface of *L. plantarum*

Approximately 5 × 10^8^ bacterial cells determined by colony forming unit were harvested by centrifugation and washed three times with cold PBS. The bacteria were resuspended in 300 µL PBS containing 1% bovine serum albumin (PBS-B) and 10 µL of monoclonal Anti-HA–FITC antibody (1 mg/mL). After incubation at 4°C for 30–60 min, the bacteria were centrifuged at 7,000×*g* for 2 min at 4°C and washed three times with 500 µL ice-cold PBS. The bacteria were subsequently fixed in 100 µL of 2% paraformaldehyde (VWR) in water for 30–60 min at 4°C. Bacteria were collected by centrifugation at 7,000×*g* for 2 min, washed three times with 500 µL PBS-B and resuspended in PBS. Fluorescent staining of resuspended bacteria was analyzed by flow cytometry using a MACSQuant analyzer (Miltenyi Biotec GmbH, Bergisch Gladbach, Germany), following the manufacturer’s instructions.

### Preparation of human dendritic cells (hDCs) and FACS analysis

Human peripheral leukocytes were prepared from blood provided by healthy volunteers according to institutional guidelines (Østfold Hospital Trust). Blood of four different donors was used. Blood was mixed with an equivalent volume of sterile PBS and half a volume of Lymphoprep (Stemcell technologies), and centrifuged for 25 min at 1,500×*g*. Lymphocyte obtained with the gradient were extracted and washed three times with PBS with 10 min of centrifugation at 1,000×*g* after each step. The last centrifuge was performed at 700×*g* to withdraw plates. The purified peripheral leukocytes were stored in 50% RPMI 1640 medium (PAA Laboratories) and 50% dimethylsulfoxide (Sigma) in liquid nitrogen.

Human peripheral leukocytes were sorted using a Macs separator and Macs CD14 plus microbeads (Miltenyi Biotec) according to the supplier’s instructions. CD14+ cells were grown for 6 days in RPMI 1640 medium (PAA Laboratories GmbH) supplemented with 1 mM sodium pyruvate, 50 mM thioglycerol, 25 µg/mL gentamicin (Garamycin), 10% (m/v) fetal calf serum (Gibco Life Technologies), 25 µg/mL of IL-4 (R&D) and 50 ng/mL of human granulocyte macrophage colony-stimulating factor (GMCSF) (Abcam). Cells were grown in a humidified incubator at 37°C and 5% CO_2_ for 6 days.

Number and size of hDCs cells were evaluated by incubation with mouse anti-human CD86 Alexa Fluor^®^700-conjugated antibody (BD Biosciences) for 1 h at room temperature protected from light. After incubation cells were washed twice with PBS and analyzed by flow cytometry using a MACSQuant analyzer (Miltenyi Biotec GmbH), following the manufacturer’s instructions. Proportion above 90% of CD86 positive cells were routinely obtained.

### Preparation of bone marrow dendritic cells (BMDCs) and FACS analysis

Bone marrow cells from 6 weeks Balb/c female mice (Janvier) were extracted from leg bones with a 25G × 5/8 needle (Terumo). Red blood cells were eliminated by treatment with red blood cell lysis buffer (Sigma) at room temperature, following the manufacturer’s protocol. After centrifugation, the cells were washed three times with RPMI1640 (PAA Laboratories). The cells were then resuspended and grown for 10 days in RPMI 1640 medium containing 1 mM sodium pyruvate, 50 mM thioglycerol, 25 µg/mL penicillin streptomycin (Garamycin), 10% fetal calf serum (Gibco Life Technologies), and 50 ng/mL mouse GMCSF (Biolegend), in a humidified incubator at 37°C with 5% CO_2_.

Number and size of BMDCs cells were evaluated by incubation with alexa-fluor-488 anti-CD86 (Biolegend) and R-PE conjugated Monoclonal antibody specific to Mouse CD11c (invitrogen). Marking was analyzed by flow cytometry using a MACSQuant analyzer (Miltenyi Biotec), following the manufacturer’s instructions. Proportion above 90% of CD86 and CD11c positive cells were routinely obtained.

### Internalization assay

1 × 10^8^ bacteria determined by colony forming unit suspended in 100 µL 1 M Tris buffer, pH 8.5, were co-incubated with 1 × 10^5^ BMDCs or hDCs (multiplicity of infection = 200) for 2 h in 500 µL RPMI 1640 medium without antibiotics, in 5% CO_2_ at 37°C. After incubation, the cells were washed three times with 1 mL PBS and incubated in 500 µL RPMI 1640 containing 150 µg/mL of gentamicin for 2 h at 37°C with 5% CO_2_, in order to kill non-internalized bacteria. Subsequently, DCs were lysed in 100 µL PBS with 2% Triton X-100 (Sigma). The lysates, containing internalized bacteria, were plated on MRS plates and colony forming units (CFU) were counted. Experiments were repeated three times, each time on three different mice for BMDCs. For hDCs, were repeated also three times the experiment but on the same four different blood donors.

In competition experiments, the hDCs were pre-incubated for 1 h with monoclonal anti-human CD205 Antibody [Purified anti-human CD205 (DEC-205) Antibody; Biolegend] at a final concentration of 2 µg/mL, prior to addition of the bacteria.

### Plasmid delivery to DCs

BMDCs or hDCs were co-incubated with the different bacterial strains as described above. After incubation, the cells were washed three times with RPMI and grown for 36 h in RPMI 1640 medium (PAA Laboratories GmbH) containing 1 mM sodium pyruvate, 50 mM thioglycerol, 25 µg/mL penicillin streptomycin (Garamycin), 10% fetal calf serum (Gibco Life Technologies). Thereafter approximately 1 × 10^6^ harvested cells were washed three times with cold PBS and them resuspended in 300 µL PBS-B. GFP expression was analyzed by flow cytometry using a MACSQuant analyzer (Miltenyi Biotec GmbH), following the manufacturer’s instructions. Experiments were repeated three times, each time on three different mice for BMDCs. For hDCs, were repeated also three times the experiment but on the same four different blood donors.

### Experimental protocol for animal experiments

Groups of 6 C57BL/6 mice (Janvier) were fed by gavage during 4 days with 1 × 10^9^ bacteria/day in 100 µL PBS. At day 4, the mice were euthanized by cervical elongation, the intestine and colon were withdrawn, opened longitudinally, and cut into 5 mm pieces which were washed 4 times by incubation for 20 min at 37°C in 5 mL PBS containing 2 mM EDTA, with strong shaking (>250 rpm). After each incubation, the samples were centrifuged at 500×*g* for 5 min and the supernatant, containing enterocytes, was discarded. Then, the fragments were incubated three times for 45 min at 37°C with strong shaking (>250 rpm) in 100U/mL of collagenase D (Roche), 30 µg DNase 1 (Sigma), 20% v/v fetal calf serum (Gibco Life Technologies) in PBS. The samples were centrifuged at 500×*g* for 5 min after each incubation and supernatants were collected, pooled and filtered with a 70 µm Falcon cell strainer (Falcon). The extracted cells were sorted with a Macs separator using mouse CD11c beads (Miltenyi Biotec) to collect DCs, according to the manufacturer’s instructions. Marking of the mouse DCs thus isolated was analyzed by flow cytometry using a MACSQuant analyzer (Miltenyi Biotec GmbH), following the manufacturer’s instructions.

### Statistical tests

Statistical significance was tested using the Mann–Whitney test on Prism (GraphPad software). Results are presented as mean ± SEM. Statistical significance was considered for p < 0.05. * is p < 0.05, ** is p < 0.01 and *** is p < 0.001.

## Abbreviations

aDec: anti-Dec-205 recombinant antibody
BLG: β-lactoglobulin
BMDC: bone marrow dendritic cell
CFU: colony forming units
CWA: cell wall anchor
DC: dendritic cell
FnBPA: fibronectin binding protein A
GIT: gastro-intestinal tract
HA: human influenza hemagglutinin
hDC: human dendritic cell
IBD: inflammatory bowel disease
InlA: internalin A
LAB: lactic acid bacteria
Lip: lipobox anchor
LysM: LysM anchor
PRR: pattern recognition receptor
ScFv: single chain Fv fragment
