# Erratum to: Surface display of an anti-DEC-205 single chain Fv fragment in *Lactobacillus plantarum* increases internalization and plasmid transfer to dendritic cells in vitro and in vivo

**DOI:** 10.1186/s12934-015-0366-6

**Published:** 2015-11-17

**Authors:** Christophe Michon, Katarzyna Kuczkowska, Philippe Langella, Vincent G. H. Eijsink, Geir Mathiesen, Jean-Marc Chatel

**Affiliations:** INRA, UMR1319 MICALIS, Bat 440, R-2, 78352 Jouy-en-Josas, France; AgroParisTech, UMR MICALIS, 78352 Jouy-en-Josas, France; Department of Chemistry, Biotechnology and Food Science, Norwegian University of Life Sciences, Aas, Norway

## Erratum to: Microb Cell Fact (2015) 14:95 DOI 10.1186/s12934-015-0290-9

Unfortunately, the original version of this article [1] contained an error. The name of the author was incorrectly written as Michon Christophe. The correct writing is Christophe Michon.
